# A lysosome-targeted dextran-doxorubicin nanodrug overcomes doxorubicin-induced chemoresistance of myeloid leukemia

**DOI:** 10.1186/s13045-021-01199-8

**Published:** 2021-11-08

**Authors:** Yunxin Zeng, Xinyu Zhang, Dongjun Lin, Xiaohui Feng, Yuye Liu, Zhengwen Fang, Weijian Zhang, Yu Chen, Meng Zhao, Jun Wu, Linjia Jiang

**Affiliations:** 1 Department of Hematology, the Seventh Affiliated Hospital of Sun Yat-Sen University, Shenzhen, China; 2 Guangdong Provincial Key Laboratory of Malignant Tumor Epigenetics and Gene Regulation, Sun Yat-Sen Memorial Hospital, Sun Yat-Sen University, Guangzhou, China; 3 School of Biomedical Engineering, Sun Yat-Sen University, Shenzhen, China; 4 Key Laboratory of Stem Cells and Tissue Engineering, Zhongshan School of Medicine, Sun Yat-Sen University, Ministry of Education, Guangzhou, China

**Keywords:** Myeloid leukemia, Chemotherapy, Doxorubicin, Hypoxia, Lysosome

## Abstract

The hypoxic microenvironment is presumed to be a sanctuary for myeloid leukemia cells that causes relapse following chemotherapy, but the underlying mechanism remains elusive. Using a zebrafish xenograft model, we observed that the hypoxic hematopoietic tissue preserved most of the chemoresistant leukemic cells following the doxorubicin (Dox) treatment. And hypoxia upregulated TFEB, a master regulator of lysosomal biogenesis, and increased lysosomes in leukemic cells. Specimens from relapsed myeloid leukemia patients also harbored excessive lysosomes, which trapped Dox and prevented drug nuclear influx leading to leukemia chemoresistance. Pharmaceutical inhibition of lysosomes enhanced Dox-induced cytotoxicity against leukemic cells under hypoxia circumstance. To overcome lysosome associated chemoresistance, we developed a pH-sensitive dextran-doxorubicin nanomedicine (Dex-Dox) that efficiently released Dox from lysosomes and increased drug nuclear influx. More importantly, Dex-Dox treatment significantly improved the chemotherapy outcome in the zebrafish xenografts transplanted with cultured leukemic cells or relapsed patient specimens. Overall, we developed a novel lysosome targeting nanomedicine that is promising to overcome the myeloid leukemia chemoresistance.

**Supplementary Information:**

The online version contains supplementary material available at 10.1186/s13045-021-01199-8.

## To the Editor

The mechanism(s) of how hypoxia regulates chemoresistance remains unclear, and the potential targeting therapeutic strategy is poorly developed [1, 2]. The zebrafish is an elegant model to investigate the efficacy of anti-leukemic drugs and the interaction between tumor and microenvironment in vivo [3–5]. Here, using the zebrafish xenograft model, we identified the hypoxic caudal hematopoietic tissue (CHT) were enriched with lysosome-abundant chemoresistant leukemic cells and further developed a lysosome targeting nanomedicine to enhance the chemotherapy efficacy.

**Fig. 1:**
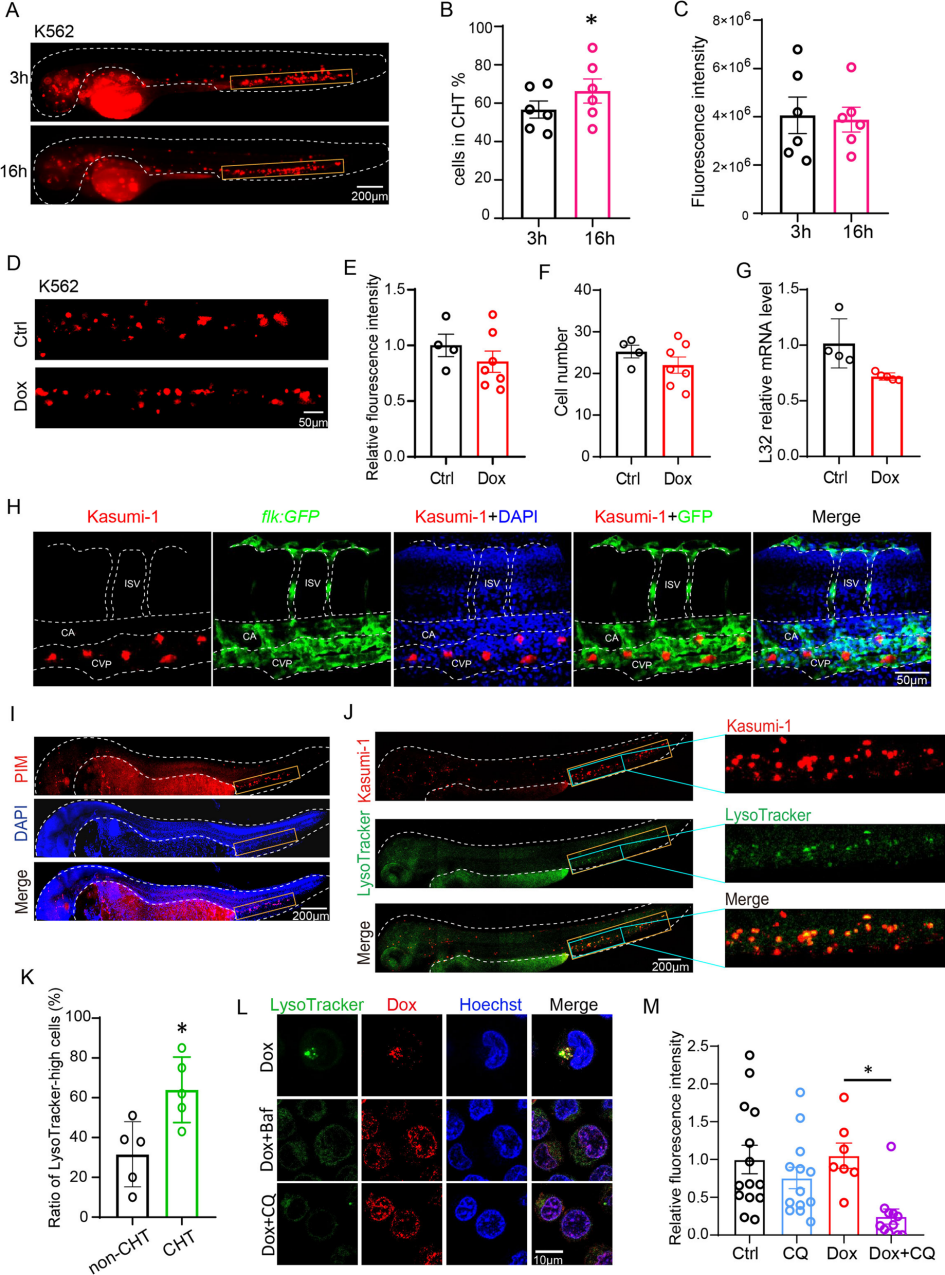
Hypoxic CHT harbored chemoresistant leukemic cells with increased lysosomes that sequestered Dox to prevent its nuclear entry and cytotoxicity. **A**–**C** K562 cells were microinjected into 2dpf embryos. The cell number and fluorescent intensity of leukemic cells localized in CHT at different time points post-injection were counted. **D**–**G** K562-xenografted zebrafish were treated with Dox from one-day-post injection (1 dpi) to 3 dpi, and the leukemic cells in CHT were quantified with the fluorescent intensity (**E**), the cell number (**F**) and the mRNA expression of human ribosome gene L32 (**G**). **H** Dox-resistant Kasumi-1 cells were mainly localized in the CVP vessels visualized by *flk:GFP* fish. CVP-caudal vein plexus, CA-caudal aorta, ISV-intersegmental vessel. **I** The 2dpf zebrafish embryos were stained with the PIM antibody to detect hypoxia tissue. The CHT was labeled with yellow rectangles. **J**, **K** The Kasumi-1-xenografted zebrafish were stained with LysoTracker and quantified for lysosome enrichment in CHT and non-CHT in vivo. The entire CHT in the left panel was labeled with yellow rectangles, and the region in blue rectangles were magnified in the right panels to show details. **L** The subcellular localization of Dox in K562 cells was visualized by its autonomous red fluorescence in Dox, Dox + Baf or Dox + CQ. **M** The chemoresistant K562 cells that localized in CHT were more sensitive to Dox + CQ while CQ alone has no toxicity. Bar plots are shown as average ± SEM. The statistical significance between groups was determined using Student’s *t*-test or ANOVA analysis. *Indicates *p* < 0.05; **p < 0.01; ***p < 0.001; ****p < 0.0001

The two days post-fertilization (2dpf) zebrafish embryos are immunodeficient due to the absence of adaptive immune system [4] and were used for xenografting myeloid leukemia cells, including Kasumi-1, K562 and OA3, to investigate the chemoresistance mechanism. The accumulated leukemic cells in CHT increased from 3-h post-injection (hpi) to 16 hpi, but the total leukemic cell number were comparable (Fig. 1A–C, Additional file 1: Fig. S1A–F). Moreover, the CHT-localized leukemic cells were mainly distributed in the caudal vein plexus of CHT (Fig. 1H). To explore the chemosensitivity of leukemic cells in CHT, we treated K562- (Fig. 1D–G) and Kasumi-1-(Additional file 1: Fig. S1G–J) xenografted zebrafish with Dox. The fluorescence intensity, cell number and the expression of human ribosome gene L32 did not significantly reduce upon Dox treatment. The leukemia cells resided in CHT were negative with the apoptosis marker TUNEL, confirming that the cells were chemoresistant (Additional file 1: Fig. S1K, L).

We then tested the 2dpf zebrafish embryos for hypoxia markers, and found the hypoxia indicator pimonidazole (PIM) and the hypoxia-associated genes *hif1al* were highly enriched in CHT (Fig. 1I and Additional file 2: Fig. S2A). Besides the lysosome-related genes TFEB, LAMP1 and LC3B also increased in K562 under hypoxia (Additional file 2: Fig. S2B). TFEB is a master regulator of lysosome biogenesis [6, 7], we then assumed that hypoxia might increase TFEB expression to activate lysosome biogenesis. Indeed, we found the lysosome-high cells and expressions of lysosome genes such as V-ATPase, LAMP1 and LAMP2 were highly enriched in hypoxic K562 and Kasumi-1 cells (Additional file 2: Fig. S2C–H). Furthermore, more CHT-localized leukemic cells were stained positive with LysoTracker compared with cells in other tissues of leukemia-zebrafish xenografts (Fig. 1J, K), indicating the hypoxic CHT preserved leukemic cells with enriched lysosomes.

We next explored the role of lysosome in regulating leukemia chemoresistance. Lysosome inhibitor bafilomycin (Baf) or chloroquine (CQ) efficiently decreased the ratio of LysoTracker- or LysoSensor-high K562 cells (Additional file 3: Fig. S3A–D). We examined the intracellular location of Dox using its autonomous red fluorescence. Dox was mainly located in lysosomes but transported into the nucleus when treated with Baf or CQ (Fig. 1L). Baf or CQ also enhanced the Dox-induced cytotoxicity against chemoresistant cells in hypoxia-cultured cells (Additional file 3: Fig. S3E) and in xenografted zebrafish (Fig. 1M, Additional file 3: Fig. S3F).

**Fig. 2:**
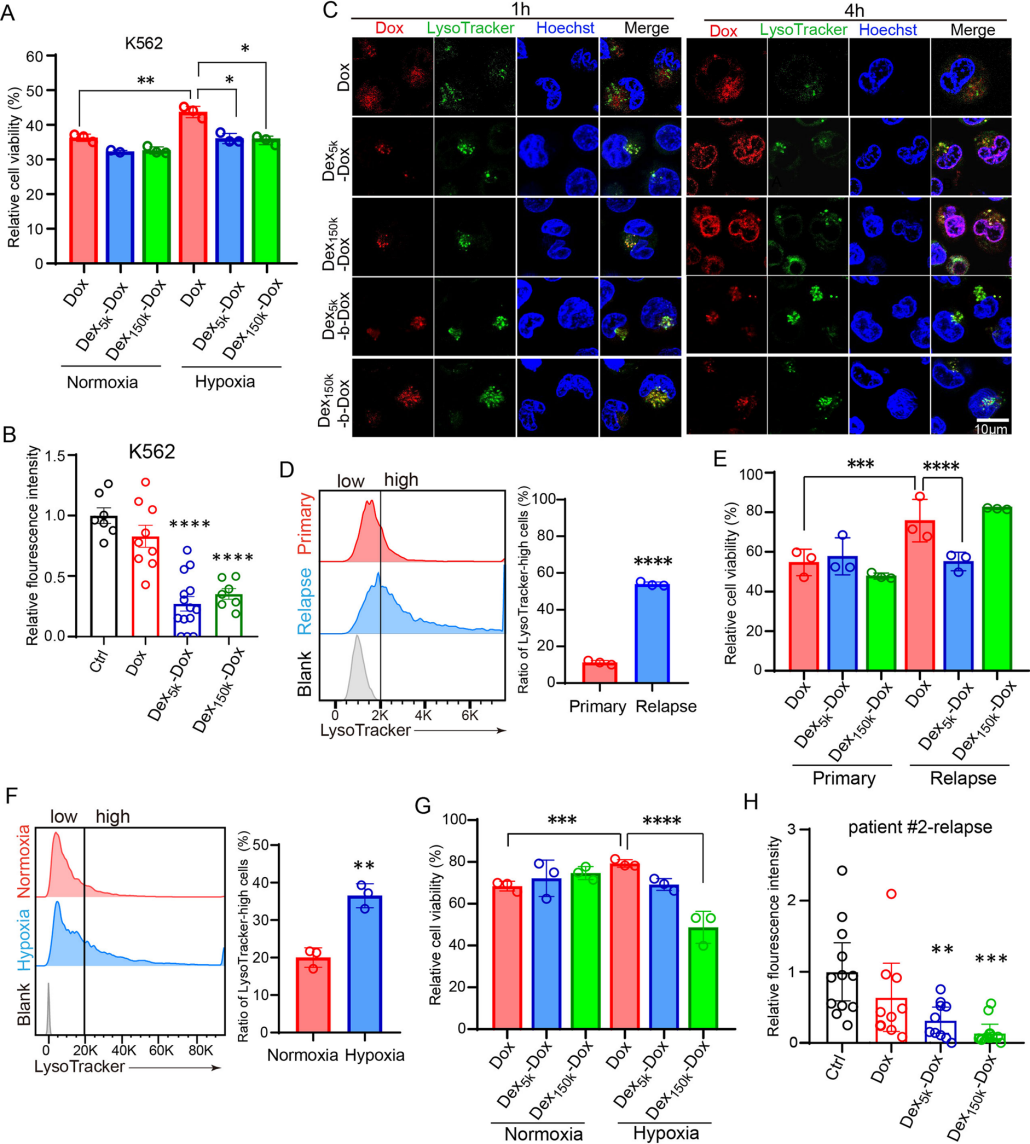
The pH-sensitive Dex-Dox nanomedicine promoted the nuclear entry and cytotoxicity of Dox in hypoxic leukemic cells to overcome chemoresistance. **A** The hypoxia-cultured K562 cells have higher viability post Dox treatment, but the viability was significantly reduced in Dex_5k/150k_-Dox. **B** The K562-xenografted-zebrafish embryos were treated with Dox or Dex_5k/150k_-Dox, and leukemic cells in CHT were counted at two days post-treatment. The results showed that Dex_5k/150k_-Dox had much higher toxicity against chemoresistant leukemic cells. **C** The subcellular localization of Dox was analyzed by its autonomous red fluorescence in Dox, Dex-Dox or Dex-b-Dox treated K562 cells. **D**, **E** The bone marrow specimen from the same patient (#1) at primary or relapsed stage were collected for staining with LysoTracker (**D**) or cell counting after treatment with Dox or Dex_5k/150k_-Dox (**E**). **F**, **G** The bone marrow specimen from the relapsed patient #2 was collected and incubated in hypoxia to measure the ratio of LysoTracker-high cells (**F**) and count the cell number after treatment with Dox or Dex_5k/150k_-Dox (**G**). **H** The zebrafish embryos were xenografted with the relapsed leukemic cells from patient #2 and treated with Dox or Dex_5k/150k_-Dox. The fluorescent intensity of leukemic cells in CHT was quantified at two days post-treatment. Bar plots are shown as average ± SEM. The statistical significance between groups was determined using Student’s *t*-test or ANOVA analysis. * Indicates *p* < 0.05; **p < 0.01; ***p < 0.001; ****p < 0.0001

Although our results showed that lysosome inhibition promotes the Dox nuclear entry and cytotoxicity against chemoresistant leukemic cells, CQ failed to improve the leukemia treatment outcome clinically due to the toxic effect and low delivery efficiency [6–10]. Therefore, we developed the lysosome targeting Dex-Dox nanodrug in which Dox was covalently conjugated with polymerized dextran  (Dex) to evaluate the anti-leukemia effect. The drug release experiment showed that the acid-responsive-bond containing Dex_5k/150k_-Dox were more efficient in releasing Dox at low pH than their negative control Dex_5k/150k_-b-Dox (Additional file 4: Fig. S4G). The results of in vitro cell viability (Additional file 5: Fig. S5A, B), apoptosis (Additional file 5: Fig. S5C, D) and ROS levels (Additional file 5: Fig. S5E, F) showed that Dex_5k/150k_-Dox had comparable cytotoxicity with Dox by eliminating normoxia-cultured Kasumi-1 or K562 cells. However, Dex_5k/150k_-Dox significantly decreased cell viability than Dox in hypoxic K562 (Fig. 2A) and Kasumi-1 (Additional file 5: Fig. S5G). In leukemia-xenografted-zebrafish, Dex_5k/150k_-Dox but not Dox remarkably eliminated chemoresistant K562 and Kasumi-1 cells in CHT (Fig. 2B and Additional file 5: Fig. S5H). Increasing lysosome pH with CQ attenuated the amplified cytotoxicity of Dex_5k_-Dox (Additional file 5: Fig. S5I), indicating Dex-Dox depends on lysosome for exerting cytotoxicity.

Mechanistically Dex-Dox induced apoptosis in chemoresistant leukemia cells as we found more TUNEL + K562 cells in Dex_5k_-Dox treated CHT (Additional file 6: Fig. S6A, B). Futhermore, Dex_5k/150k_-Dox released Dox from lysosomes to enter the nuclei (Fig. 2C), but had no effect on lysosome pH as compared to Dox (Additional file 6: Fig. S6C–H), suggesting Dex_5k/150k_-Dox might exhibit anti-leukemic effect through facilitating Dox nuclear influx. In addition, Dox released from Dex-Dox nanomedicine was highly accumulated in zebrafish and transported into the CHT localized leukemic cells more efficiently than Dox alone (Additional file 7: Fig. S7A–D).

We further explored the therapeutic effect of Dex-Dox with myeloid leukemia patient samples. The leukemic cells from the relapsed patient had increased ratio of LysoTracker-high cells than the primary patient cells (Fig. 2D), and Dex_5k_-Dox efficiently eliminated the relapsed cells than Dox (Fig. 2E). Similarly, the hypoxia-incubated leukemic cells had increased ratio of LysoTracker-high cells, and more resistant to Dox, but they were susceptible to Dex_150k_-Dox treatment (Fig. 2F, G). We also found that Dex_5k/150k_-Dox efficiently eliminated these relapsed patient cells in xenografted zebrafish (Fig. 2H, Additional file 7: Fig. S7E, F).

Overall, our data reveal that the hypoxia-lysosome axis controls the myeloid leukemia chemoresistance, and the newly developed lysosome targeting nanomedicine is a promising strategy to eliminate chemoresistant leukemic cells (Additional file 8: Fig. S8).

## Supplementary Information

**Additional file 1**.** Figure S1. Chemoresistant leukemic cells mainly resided in the caudal hematopoietic tissue (CHT) of xenografted zebrafish.** (A–F) Kasumi-1 or OA3 cells were microinjected into 2dpf embryos and the fluorescent intensities of leukemic cells localized in CHT (highlighted by the yellow box) and non-CHT at different time points post-injection were quantified. Some cells entered the yolk sac during microinjection and were excluded for counting. (G–J) Kasumi-1 xenografted zebrafish were treated with Dox at one day post-injection (1dpi), and after two days the leukemic cells in CHT were quantified for the fluorescent intensity (H), the cell number (I) and the mRNA expression of human ribosome gene L32 (J). (K–L) The DiI-labeled Kasumi-1 cells were xenografted into zebrafish embryos and treated with Dox before staining with TUNEL to identify the apoptotic cells. (K) The TUNEL+DiI+ cells in CHT and non-CHT were quantified and the ratio was calculated by dividing the total DiI+ cell number.

**Additional file 2**.** Figure S2. Hypoxic microenvironment characterized leukemic cells with enriched lysosomes.** (A) The expressions of hypoxia-associated genes hif1al, hif1ab in 2dpf embryos and hematopoietic specific marker cmyb were detected by in situ hybridization. The CHT region was highlighted with yellow box. (B) The hypoxia-cultured K562 cells were examined by Western blot for expressions of lysosome-related genes LC3B, TFEB, LAMP1. (C–E) The hypoxia-cultured K562 cells were examined for the ratio of LysoTracker-high cells (C–D) and mRNA levels of lysosome-associated genes V-ATPase, LAMP1, LAMP2 (E). (F–H) The ratio of LysoTracker-high cells (F–G) and the lysosome gene expression (H) were significantly increased in hypoxic Kasumi-1 cells compared with normal condition.

**Additional file 3**.** Figure S3. Excessive lysosome prevents the Dox cytotoxicity.** (A–B) K562 cells were treated with V-ATPase inhibitor bafilomycin (Baf) or lysosome inhibitor chloroquine (CQ). The ratio of LysoTracker high cells was quantified by flow cytometry. (C–D) K562 cells were treated Baf or CQ, and the ratio of LysoSensor high cells was quantified by flow cytometry. (E) The hypoxia-treated K562 cells have higher viability following Dox treatment, but the viability was significantly reduced in CQ+Dox or Baf+Dox. (F) K562 cells were labeled with DiI and xenografted into zebrafish embryos before treating with CQ, Dox or both. The K562 cells in CHT were quantified by fluorescent intensity at 48h post treatment.

**Additional file 4**.** Figure S4. Characterization of Dex-Dox/Dex-b-DOX nanoparticles.** (A-B) We synthesized the oxidized dextran (Dex-CHO) with dextran of different molecular weights (Dex_5k/20K/70K/150K_) and then conjugated with Dox. As a negative control, we reduced the pH-sensitive imine bond (-C=N-) in Dex-Dox to obtain the pH-insensitive carbon-nitrogen bond (-C-N-) in Dex-b-Dox. (A) The peak at 9.6 ppm in 1H NMR results proved the successful synthesis of Dex-CHO. In Dex-Dox or Dex-b-Dox the benzene peaks of Dox appeared at around 7.8 ppm while the Dex-CHO peak at 9.6 ppm disappeared, which proved their successful synthesis. (B) The representative peaks of Dex-Dox, Dex-b-Dox or Dex-CHO were at 1640 cm^-1^(*v-C=N-*), 1210 cm^-1^(*v-C-N-*) or 1720 cm^-1^(*v-C=O-*) in the Fourier transform infrared (FT-IR) spectrum. (C) In dynamic light scattering (DLS) results the particle size of Dex_5k/150k_-Dox or Dex_5k/150k_-b-Dox was respectively 35 nm or 60 nm, both with the small polymer dispersity index (PDI). (D–E) Dex_5k_, Dex_40k_, Dex_70k_, and Dex_150k_ have similar CHO contents, quantified by oxidation degree, and Dox drug loading (E). But Dex_20k_-Dox and Dex_70k_-Dox have relatively larger particle sizes and PDI than Dex5k-Dox and Dex1_50k_-Dox in DLS assay (D), resulting in precipitation and poor stability. Therefore, we cho se Dex5k-Dox and Dex_150k_-Dox for the follow-up experiments. (F) Typical transmitted electron microscopy (TEM) images of Dex_5k/150k_-Dox and Dex_5k/150k_-b-Dox. (G) Time and pH-dependent (pH 7.4, 6.8, 5.8, or 5.0) Dox release profiles of Dex_5k/150k_-Dox and Dex_5k/150k_-b-Dox in vitro (n = 3).

**Additional file 5**.** Figure S5. The pH-responsive Dex-Dox nanomedicine efficiently eliminate hypoxic leukemic cells in vitro.** The levels of cell viability (A, B), apoptosis (C, D) and ROS labeled by oxidative detective reagent DCFH (E, F) were measured in Dox or Dex_5k/150k_ treated K562 or Kasumi-1 cells (n = 3). (G) The hypoxia-cultured Kasumi-1 cells have higher viability post Dox treatment, but the viability was significantly reduced in Dex_5k/150k_-Dox (n = 3). (H) The Kasumi-1-xenografted-zebrafish embryos were treated with Dox or Dex_5k/150k_-Dox from 1dpi to 3dpi, and leukemic cells in CHT were counted for fluorescence intensity. (I) The K562-xenografted-zebrafish embryos were pretreated with CQ before adding Dex_5k_-Dox from 1dpi to 3dpi, and leukemic cells in CHT were quantified for fluorescence intensity.

**Additional file 6**.** Figure S6**.** Dox and Dex-Dox similarly impaired lysosome acidification of leukemia cells.** (A–B) The K562-xenografted zebrafish were treated with Dox or Dex Dex5k-Dox before staining for TUNEL. The TUNEL+DiI+ cells in CHT and non-CHT were quantified and the ratio was calculated by dividing the total DiI+ leukemia cell number. (C–D) Hypoxia-cultured Kasumi-1 cells were treated with Dox or Dex5k/150k-Dox, stained with LysoSensor DND-189, followed by imaging with microscope (C) or analyzing by flow cytometry (D). Kasumi-1 cells in normoxia or hypoxia were gated in flow results to quantify the ratio of LysoSensor-high cells (E–F). (G–H) The normoxia-cultured or hypoxia-cultured K562 cells were gated in flow results to quantify the ratio of LysoSensor-high cells.

**Additional file 7**.** Figure S7. The pH-responsive Dex-Dox efficiently accumulated to eliminate chemoresistant leukemic cells in vivo.** (A–B) The 2dpf zebrafish embryos were treated with DMSO, Dox or Dex_5k_-Dox for 24 h before imaging under microscope and the autonomous red fluorescence of Dox were quantified. (C–D) The GFP expressing Kasumi-1 cells were xenografted into zebrafish embryos and the Dox localization was imaged by red fluorescence. More Dex_5k_-Dox treated cells have red fluorescence compared with the Dox alone, suggesting Dex_5k_-Dox more efficiently delivered Dox into the CHT localized leukemia cells. (E–F) The zebrafish embryos were xenografted with the leukemic cells from two relapsed patients and treated with Dox or Dex_5k/150k_-Dox. The fluorescent intensity of leukemic cells in CHT was counted at two-day post-treatment.

**Additional file 8**.** Figure S8. Graphic abstract.** By visualizing in a zebrafish xenograft model, we found that the chemoresistant leukemic cells were mainly accumulated in the hypoxic hematopoietic tissue (A). The hypoxic microenvironment characterized leukemic cells with excessive lysosomes, thereby sequestering the chemotherapeutics inside to reduce its cytotoxicity (left panel in C). We developed the pH-sensitive Dex-Dox nanomedicine to release Dox from lysosomes and enter the nucleus, thereby efficiently eliminating the chemoresistant leukemic cells in vivo (B, right panel in C).

## Data Availability

All data generated during this study are included in this published article.

## Abbreviations

Dox: Doxorubicin
Dex-Dox: Dextran-doxorubicin
CHT: Caudal hematopoietic tissue
2dpf: Two days post-fertilization
PIM: Pimonidazole
Baf: Bafilomycin
CQ: Chloroquine

## References

[1] Zhang H, Li H, Xi HS, Li S (2012). HIF1alpha is required for survival maintenance of chronic myeloid leukemia stem cells. Blood.

[2] Yao X, Tan J, Lim KJ, Koh J, Ooi WF, Li Z, Huang D, Xing M, Chan YS, Qu JZ, Tay ST, Wijaya G, Lam YN, Hong JH, Lee-Lim AP, Guan P, Ng MSW, He CZ, Lin JS, Nandi T, Qamra A, Xu C, Myint SS, Davies JOJ, Goh JY, Loh G, Tan BC, Rozen SG, Yu Q, Tan IBH, Cheng CWS, Li S, Chang KTE, Tan PH, Silver DL, Lezhava A, Steger G, Hughes JR, Teh BT, Tan P (2017). VHL Deficiency Drives Enhancer Activation of Oncogenes in Clear Cell Renal Cell Carcinoma. Cancer Discov.

[3] Bentley VL, Veinotte CJ, Corkery DP, Pinder JB, LeBlanc MA, Bedard K, Weng AP, Berman JN, Dellaire G (2015). Focused chemical genomics using zebrafish xenotransplantation as a pre-clinical therapeutic platform for T-cell acute lymphoblastic leukemia. Haematologica.

[4] Deveau AP, Bentley VL, Berman JN (2017). Using zebrafish models of leukemia to streamline drug screening and discovery. Exp Hematol.

[5] Pruvot B, Jacquel A, Droin N, Auberger P, Bouscary D, Tamburini J, Muller M, Fontenay M, Chluba J, Solary E (2011). Leukemic cell xenograft in zebrafish embryo for investigating drug efficacy. Haematologica.

[6] Crowley LC, O'Donovan TR, Nyhan MJ, McKenna SL (2013). Pharmacological agents with inherent anti-autophagic activity improve the cytotoxicity of imatinib. Oncol Rep.

[7] Kim Y, Eom JI, Jeung HK, Jang JE, Kim JS, Cheong JW, Kim YS, Min YH (2015). Induction of cytosine arabinoside-resistant human myeloid leukemia cell death through autophagy regulation by hydroxychloroquine. Biomed Pharmacother.

[8] Auberger P, Puissant A (2017). Autophagy, a key mechanism of oncogenesis and resistance in leukemia. Blood.

[9] Pellegrini P, Strambi A, Zipoli C, Hagg-Olofsson M, Buoncervello M, Linder S, De Milito A (2014). Acidic extracellular pH neutralizes the autophagy-inhibiting activity of chloroquine: implications for cancer therapies. Autophagy.

[10] Varisli L, Cen O, Vlahopoulos S (2020). Dissecting pharmacological effects of chloroquine in cancer treatment: interference with inflammatory signaling pathways. Immunology.

