# Supplementary figures and images for: A lysosome-targeted dextran-doxorubicin nanodrug overcomes doxorubicin-induced chemoresistance of myeloid leukemia
Source: J Hematol Oncol. 2021 Nov 8;14:189. doi: 10.1186/s13045-021-01199-8 (PMC8576957; doi:10.1186/s13045-021-01199-8)

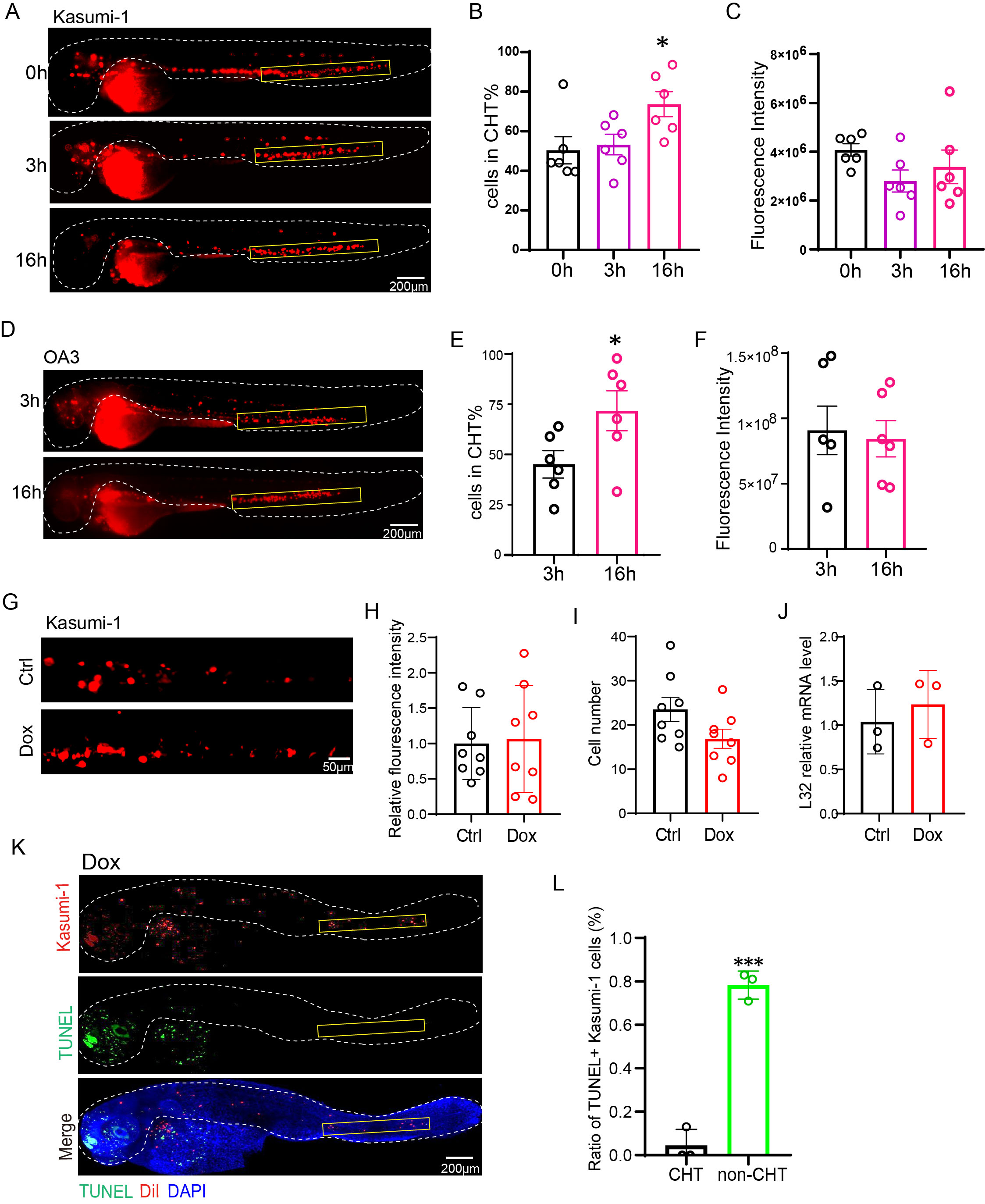

Supplement: Supplementary file 1 — Additional file 1. Figure S1. Chemoresistant leukemic cells mainly resided in the caudal hematopoietic tissue (CHT) of xenografted zebrafish. (A–F) Kasumi-1 or OA3 cells were microinjected into 2dpf embryos and the fluorescent intensities of leukemic cells localized in CHT (highlighted by the yellow box) and non-CHT at different time points post-injection were quantified. Some cells entered the yolk sac during microinjection and were excluded for counting. (G–J) Kasumi-1 xenografted zebrafish were treated with Dox at one day post-injection (1dpi), and after two days the leukemic cells in CHT were quantified for the fluorescent intensity (H), the cell number (I) and the mRNA expression of human ribosome gene L32 (J). (K–L) The DiI-labeled Kasumi-1 cells were xenografted into zebrafish embryos and treated with Dox before staining with TUNEL to identify the apoptotic cells. (K) The TUNEL+DiI+ cells in CHT and non-CHT were quantified and the ratio was calculated by dividing the total DiI+ cell number. [file 13045_2021_1199_MOESM1_ESM.jpg]

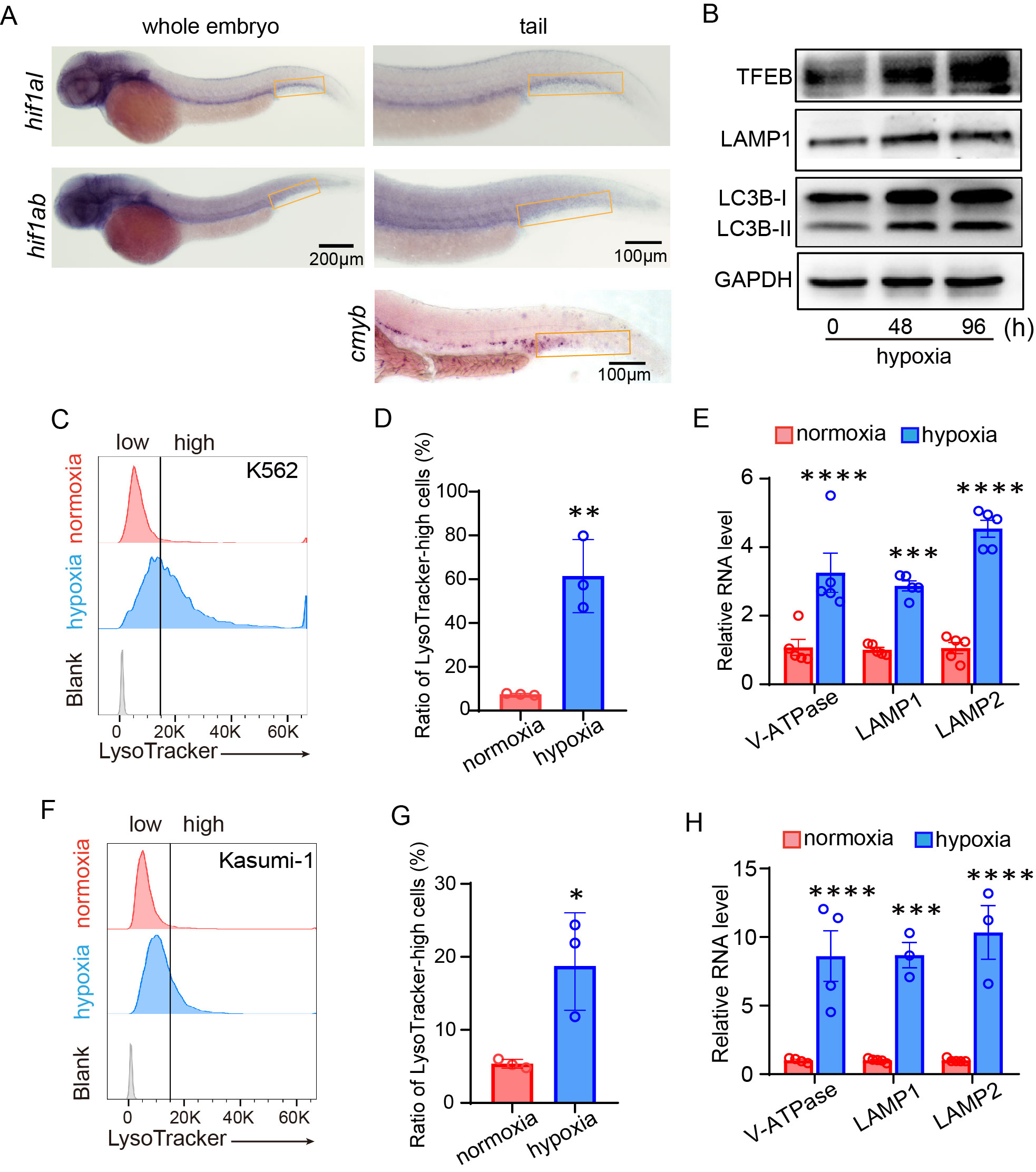

Supplement: Supplementary file 2 — Additional file 2. Figure S2. Hypoxic microenvironment characterized leukemic cells with enriched lysosomes. (A) The expressions of hypoxia-associated genes hif1al, hif1ab in 2dpf embryos and hematopoietic specific marker cmyb were detected by in situ hybridization. The CHT region was highlighted with yellow box. (B) The hypoxia-cultured K562 cells were examined by Western blot for expressions of lysosome-related genes LC3B, TFEB, LAMP1. (C–E) The hypoxia-cultured K562 cells were examined for the ratio of LysoTracker-high cells (C–D) and mRNA levels of lysosome-associated genes V-ATPase, LAMP1, LAMP2 (E). (F–H) The ratio of LysoTracker-high cells (F–G) and the lysosome gene expression (H) were significantly increased in hypoxic Kasumi-1 cells compared with normal condition. [file 13045_2021_1199_MOESM2_ESM.jpg]

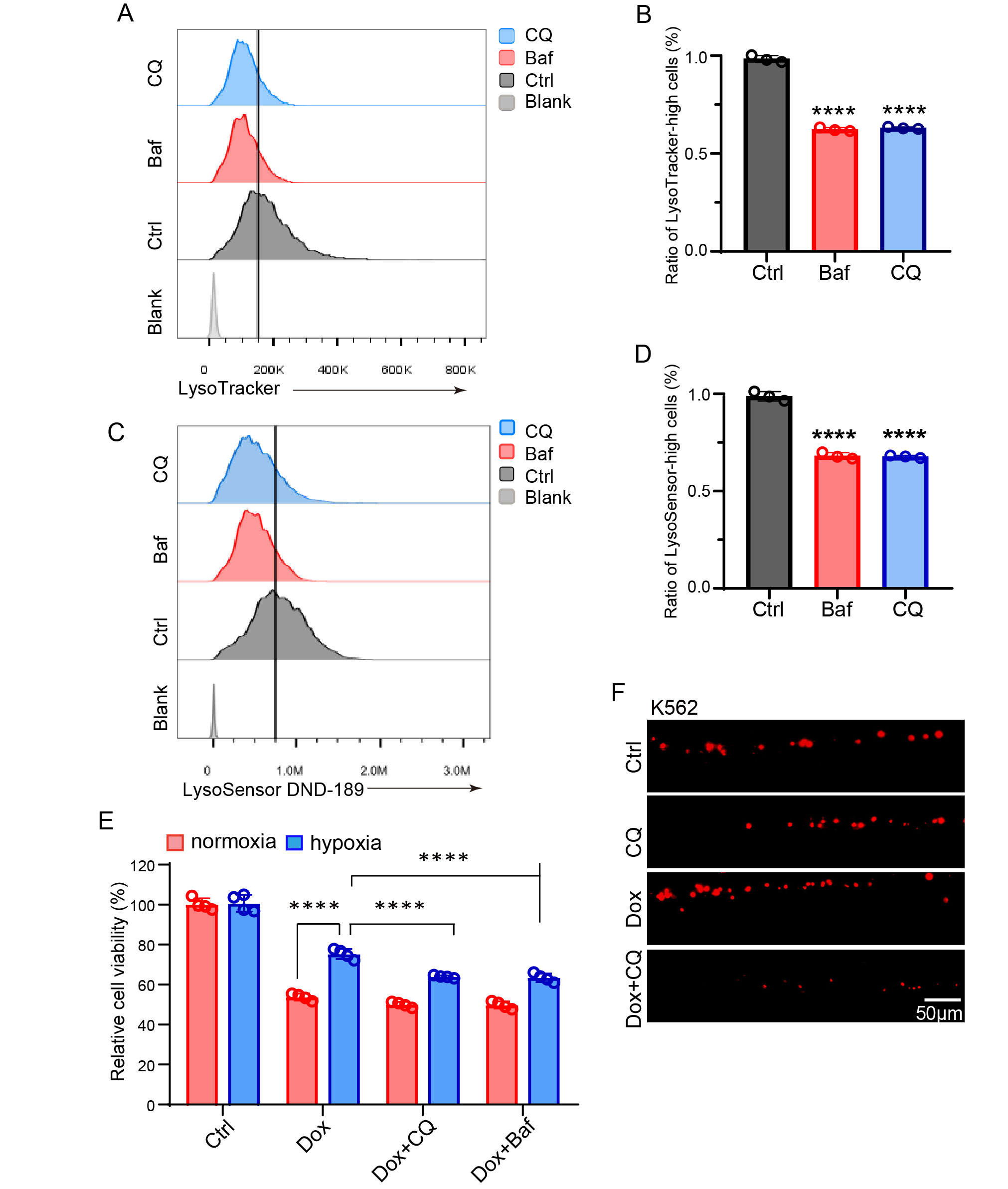

Supplement: Supplementary file 3 — Additional file 3. Figure S3. Excessive lysosome prevents the Dox cytotoxicity. (A–B) K562 cells were treated with V-ATPase inhibitor bafilomycin (Baf) or lysosome inhibitor chloroquine (CQ). The ratio of LysoTracker high cells was quantified by flow cytometry. (C–D) K562 cells were treated Baf or CQ, and the ratio of LysoSensor high cells was quantified by flow cytometry. (E) The hypoxia-treated K562 cells have higher viability following Dox treatment, but the viability was significantly reduced in CQ+Dox or Baf+Dox. (F) K562 cells were labeled with DiI and xenografted into zebrafish embryos before treating with CQ, Dox or both. The K562 cells in CHT were quantified by fluorescent intensity at 48h post treatment. [file 13045_2021_1199_MOESM3_ESM.jpg]

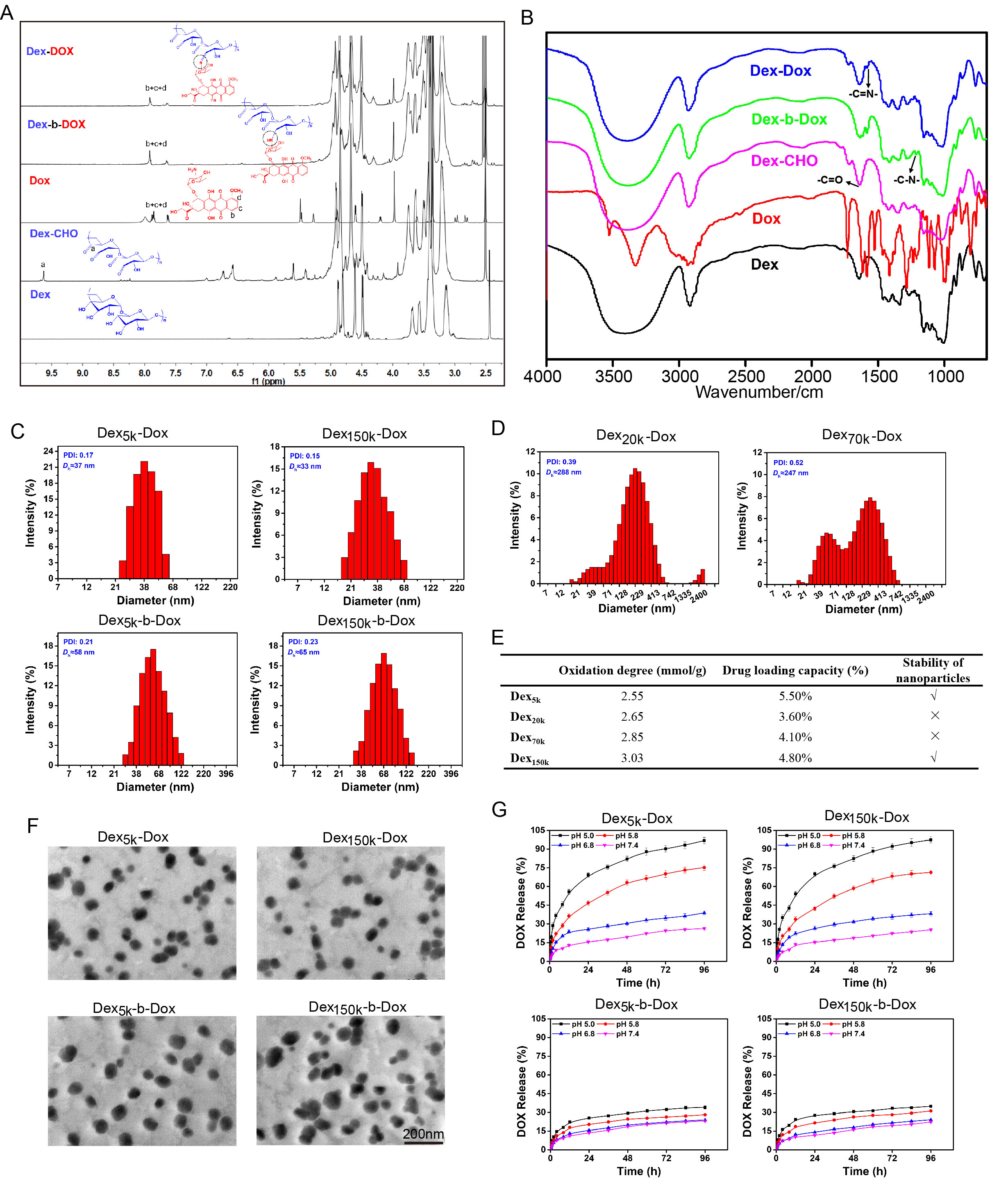

Supplement: Supplementary file 4 — Additional file 4. Figure S4. Characterization of Dex-Dox/Dex-b-DOX nanoparticles. (A-B) We synthesized the oxidized dextran (Dex-CHO) with dextran of different molecular weights (Dex5k/20K/70K/150K) and then conjugated with Dox. As a negative control, we reduced the pH-sensitive imine bond (-C=N-) in Dex-Dox to obtain the pH-insensitive carbon-nitrogen bond (-C-N-) in Dex-b-Dox. (A) The peak at 9.6 ppm in 1H NMR results proved the successful synthesis of Dex-CHO. In Dex-Dox or Dex-b-Dox the benzene peaks of Dox appeared at around 7.8 ppm while the Dex-CHO peak at 9.6 ppm disappeared, which proved their successful synthesis. (B) The representative peaks of Dex-Dox, Dex-b-Dox or Dex-CHO were at 1640 cm-1(v-C=N-), 1210 cm-1(v-C-N-) or 1720 cm-1(v-C=O-) in the Fourier transform infrared (FT-IR) spectrum. (C) In dynamic light scattering (DLS) results the particle size of Dex5k/150k-Dox or Dex5k/150k-b-Dox was respectively 35 nm or 60 nm, both with the small polymer dispersity index (PDI). (D–E) Dex5k, Dex40k, Dex70k, and Dex150k have similar CHO contents, quantified by oxidation degree, and Dox drug loading (E). But Dex20k-Dox and Dex70k-Dox have relatively larger particle sizes and PDI than Dex5k-Dox and Dex150k-Dox in DLS assay (D), resulting in precipitation and poor stability. Therefore, we cho se Dex5k-Dox and Dex150k-Dox for the follow-up experiments. (F) Typical transmitted electron microscopy (TEM) images of Dex5k/150k-Dox and Dex5k/150k-b-Dox. (G) Time and pH-dependent (pH 7.4, 6.8, 5.8, or 5.0) Dox release profiles of Dex5k/150k-Dox and Dex5k/150k-b-Dox in vitro (n = 3). [file 13045_2021_1199_MOESM4_ESM.jpg]

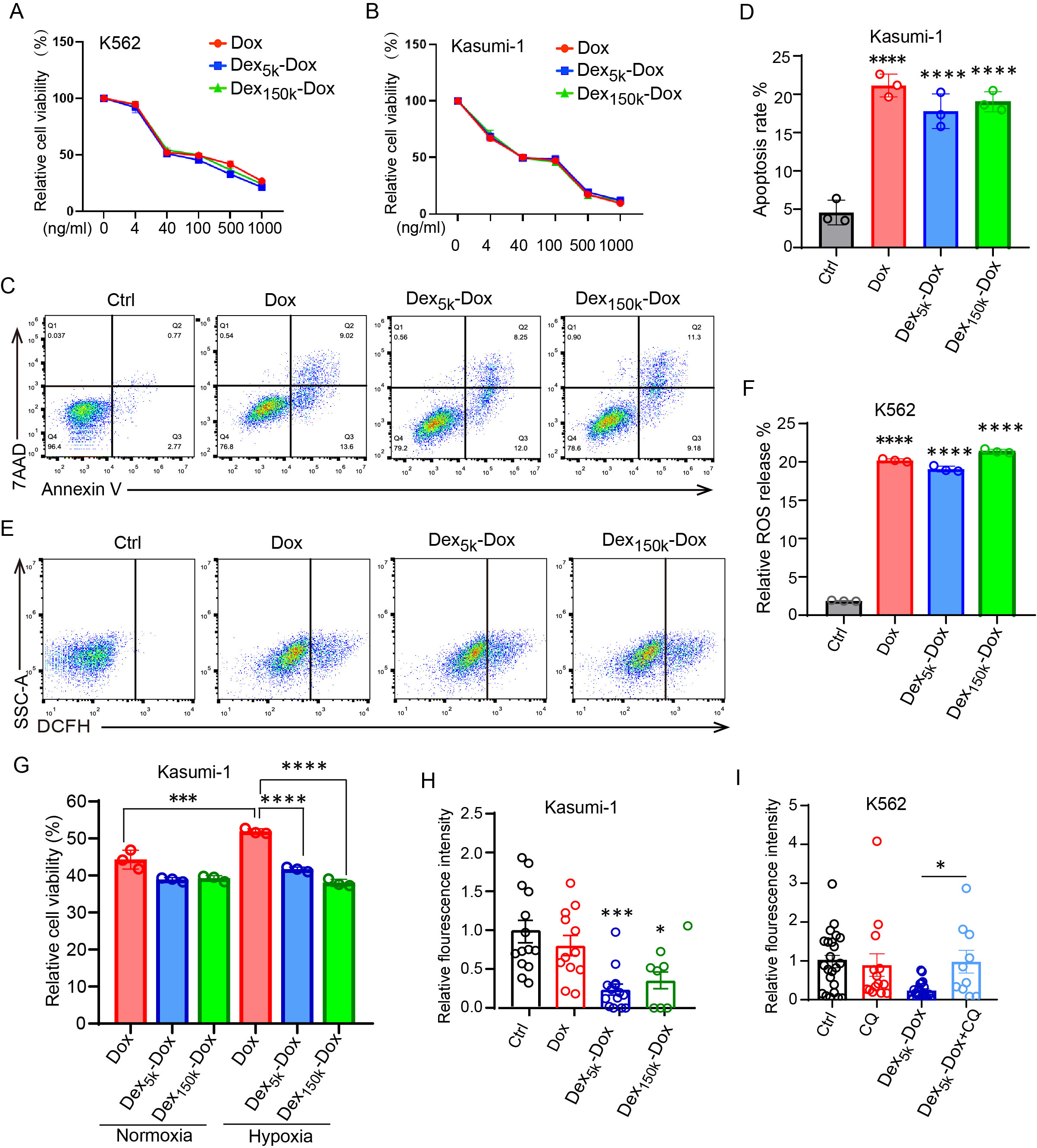

Supplement: Supplementary file 5 — Additional file 5. Figure S5. The pH-responsive Dex-Dox nanomedicine efficiently eliminate hypoxic leukemic cells in vitro. The levels of cell viability (A, B), apoptosis (C, D) and ROS labeled by oxidative detective reagent DCFH (E, F) were measured in Dox or Dex5k/150k treated K562 or Kasumi-1 cells (n = 3). (G) The hypoxia-cultured Kasumi-1 cells have higher viability post Dox treatment, but the viability was significantly reduced in Dex5k/150k-Dox (n = 3). (H) The Kasumi-1-xenografted-zebrafish embryos were treated with Dox or Dex5k/150k-Dox from 1dpi to 3dpi, and leukemic cells in CHT were counted for fluorescence intensity. (I) The K562-xenografted-zebrafish embryos were pretreated with CQ before adding Dex5k-Dox from 1dpi to 3dpi, and leukemic cells in CHT were quantified for fluorescence intensity. [file 13045_2021_1199_MOESM5_ESM.jpg]

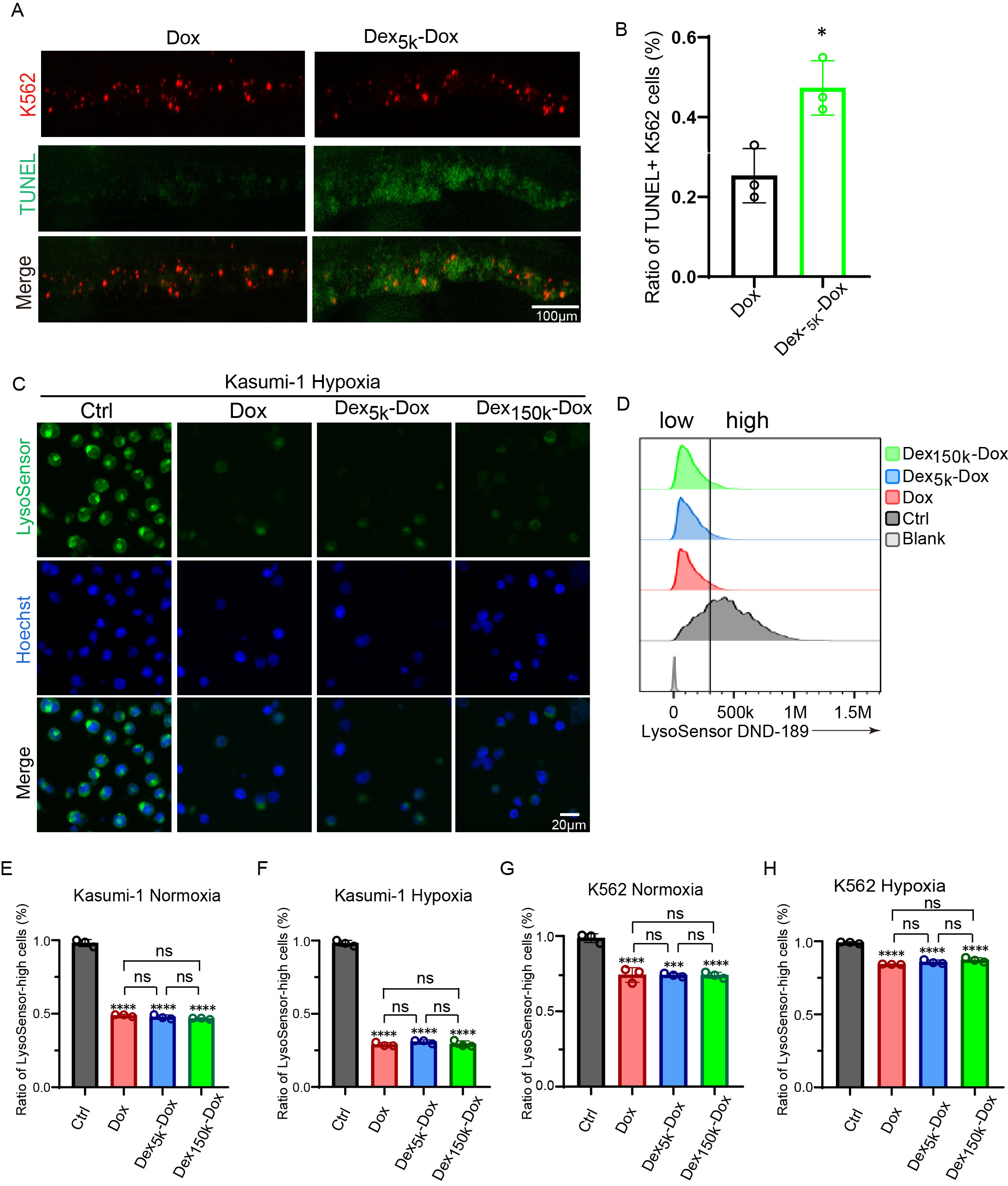

Supplement: Supplementary file 6 — Additional file 6. Figure S6. Dox and Dex-Dox similarly impaired lysosome acidification of leukemia cells. (A–B) The K562-xenografted zebrafish were treated with Dox or Dex Dex5k-Dox before staining for TUNEL. The TUNEL+DiI+ cells in CHT and non-CHT were quantified and the ratio was calculated by dividing the total DiI+ leukemia cell number. (C–D) Hypoxia-cultured Kasumi-1 cells were treated with Dox or Dex5k/150k-Dox, stained with LysoSensor DND-189, followed by imaging with microscope (C) or analyzing by flow cytometry (D). Kasumi-1 cells in normoxia or hypoxia were gated in flow results to quantify the ratio of LysoSensor-high cells (E–F). (G–H) The normoxia-cultured or hypoxia-cultured K562 cells were gated in flow results to quantify the ratio of LysoSensor-high cells. [file 13045_2021_1199_MOESM6_ESM.jpg]

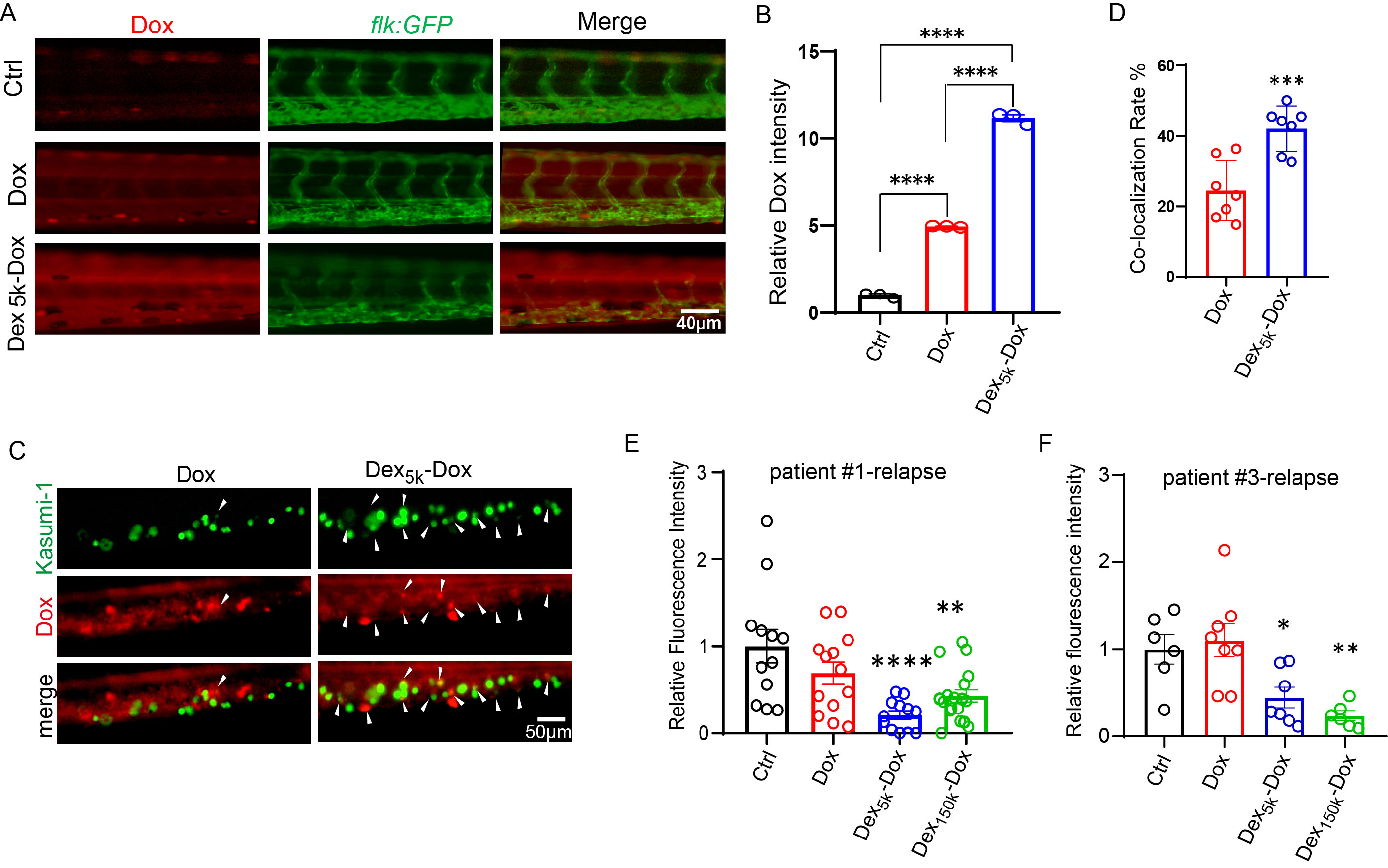

Supplement: Supplementary file 7 — Additional file 7. Figure S7. The pH-responsive Dex-Dox efficiently accumulated to eliminate chemoresistant leukemic cells in vivo. (A–B) The 2dpf zebrafish embryos were treated with DMSO, Dox or Dex5k-Dox for 24 h before imaging under microscope and the autonomous red fluorescence of Dox were quantified. (C–D) The GFP expressing Kasumi-1 cells were xenografted into zebrafish embryos and the Dox localization was imaged by red fluorescence. More Dex5k-Dox treated cells have red fluorescence compared with the Dox alone, suggesting Dex5k-Dox more efficiently delivered Dox into the CHT localized leukemia cells. (E–F) The zebrafish embryos were xenografted with the leukemic cells from two relapsed patients and treated with Dox or Dex5k/150k-Dox. The fluorescent intensity of leukemic cells in CHT was counted at two-day post-treatment. [file 13045_2021_1199_MOESM7_ESM.jpg]

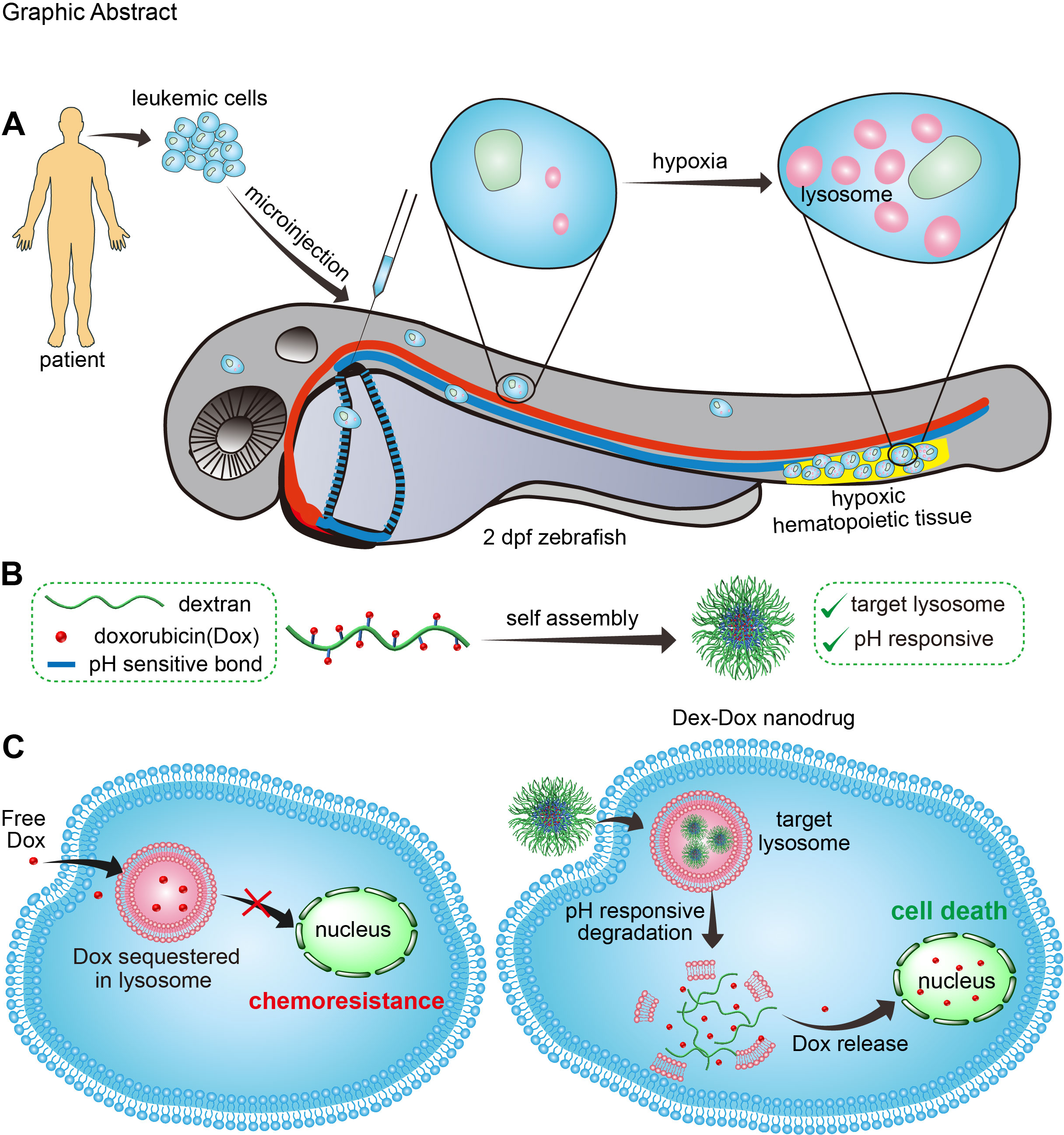

Supplement: Supplementary file 8 — Additional file 8. Figure S8. Graphic abstract. By visualizing in a zebrafish xenograft model, we found that the chemoresistant leukemic cells were mainly accumulated in the hypoxic hematopoietic tissue (A). The hypoxic microenvironment characterized leukemic cells with excessive lysosomes, thereby sequestering the chemotherapeutics inside to reduce its cytotoxicity (left panel in C). We developed the pH-sensitive Dex-Dox nanomedicine to release Dox from lysosomes and enter the nucleus, thereby efficiently eliminating the chemoresistant leukemic cells in vivo (B, right panel in C). [file 13045_2021_1199_MOESM8_ESM.jpg]
